# Editorial: P2X7 as Common Therapeutic Target in Brain Diseases

**DOI:** 10.3389/fnmol.2021.656011

**Published:** 2021-04-27

**Authors:** Tobias Engel, Annette Nicke, Jan M. Deussing, Beata Sperlagh, Miguel Diaz-Hernandez

**Affiliations:** ^1^Department of Physiology and Medical Physics, Royal College of Surgeons in Ireland, University of Medicine and Health Sciences, Dublin, Ireland; ^2^FutureNeuro, Science Foundation Ireland Research Centre for Chronic and Rare Neurological Diseases, Royal College of Surgeons in Ireland, University of Medicine and Health Sciences, Dublin, Ireland; ^3^Faculty of Medicine, Walther Straub Institute of Pharmacology and Toxicology, Ludwig-Maximilians-Universität München, Munich, Germany; ^4^Molecular Neurogenetics, Max Planck Institute of Psychiatry, Munich, Germany; ^5^Laboratory of Molecular Pharmacology, Institute of Experimental Medicine, Budapest, Hungary; ^6^Department of Biochemistry and Molecular Biology, Veterinary School, Complutense University of Madrid, Madrid, Spain; ^7^Instituto de Investigación Sanitaria del Hospital Clínico San Carlos, Madrid, Spain

**Keywords:** purinergic signaling, ATP, P2X7 receptor, brain diseases, shared pathological pathways

Despite differences in disease etiology (e.g., β-amyloid, polyglutamine expansion, or neurodevelopmental abnormalities), several brain diseases (e.g., Alzheimer's disease, epilepsy, or schizophrenia) share common clinical symptoms with overlapping diagnoses including depression, psychotic episodes, cognitive deficits, anxiety, and seizures. This implies the activation of shared pathological pathways in different brain diseases. An emerging concept is that increased hyperexcitability and network changes are universal pathomechanisms in numerous brain diseases (Palop and Mucke, 2010; Cepeda-Prado et al., 2012; Kanner, 2012; Nakahara et al., 2018). As well as neurons, glial cells are involved in network hyperexcitability and the mediation of inflammatory processes by modulating the release of neurotransmitters and pro-inflammatory cytokines (Robel and Sontheimer, 2016).

Purinergic signaling mediated *via* specific purinergic membrane receptors, which are activated by extracellularly released nucleosides (P1 adenosine receptors) and nucleotides [e.g., adenosine triphosphate (ATP)] (P2Y and P2X receptors), were suggested to play an important role in numerous human pathological conditions including diseases of the central nervous system (CNS) (Burnstock, 2020). The P2X7 receptor belongs to the ATP-gated ionotropic P2X receptor family. Among the P2X receptors, it has some unique structural and functional characteristics, that make this receptor a particularly attractive therapeutic target (Sperlagh and Illes, 2014; Jimenez-Mateos et al., 2019; Kopp et al., 2019). In particular, it has a much lower affinity for ATP [activation threshold: 0.3–0.5 mM; however, decreased activation threshold (0.05–0.1 mM) has been reported during inflammation (Di Virgilio et al., 2017)], suggesting that its activation occurs mainly under pathological conditions of high ATP release. Furthermore, it is slowly desensitizing, can induce plasma membrane permeabilization for large molecules, and is a key driver of inflammation (Di Virgilio et al., 2017). Studies have attributed a wide array of pathological processes to P2X7 receptor activation in the brain, most prominently the activation of pro-inflammatory processes and regulation of neurotransmitter release. In addition, P2X7 activation has been linked to other damaging processes such as the promotion of cell death, hyperexcitability, and permeabilization of the blood brain barrier (Sperlagh and Illes, 2014). These are shared by the majority of brain diseases and potentially contribute to both primary disease pathology and associated comorbidities (Figure 1). In support of this hypothesis, mounting data demonstrate beneficial effects of P2X7 receptor antagonism in numerous brain diseases including neurodegenerative, psychiatric, and neurological diseases (Sperlagh and Illes, 2014).

**Figure 1:**
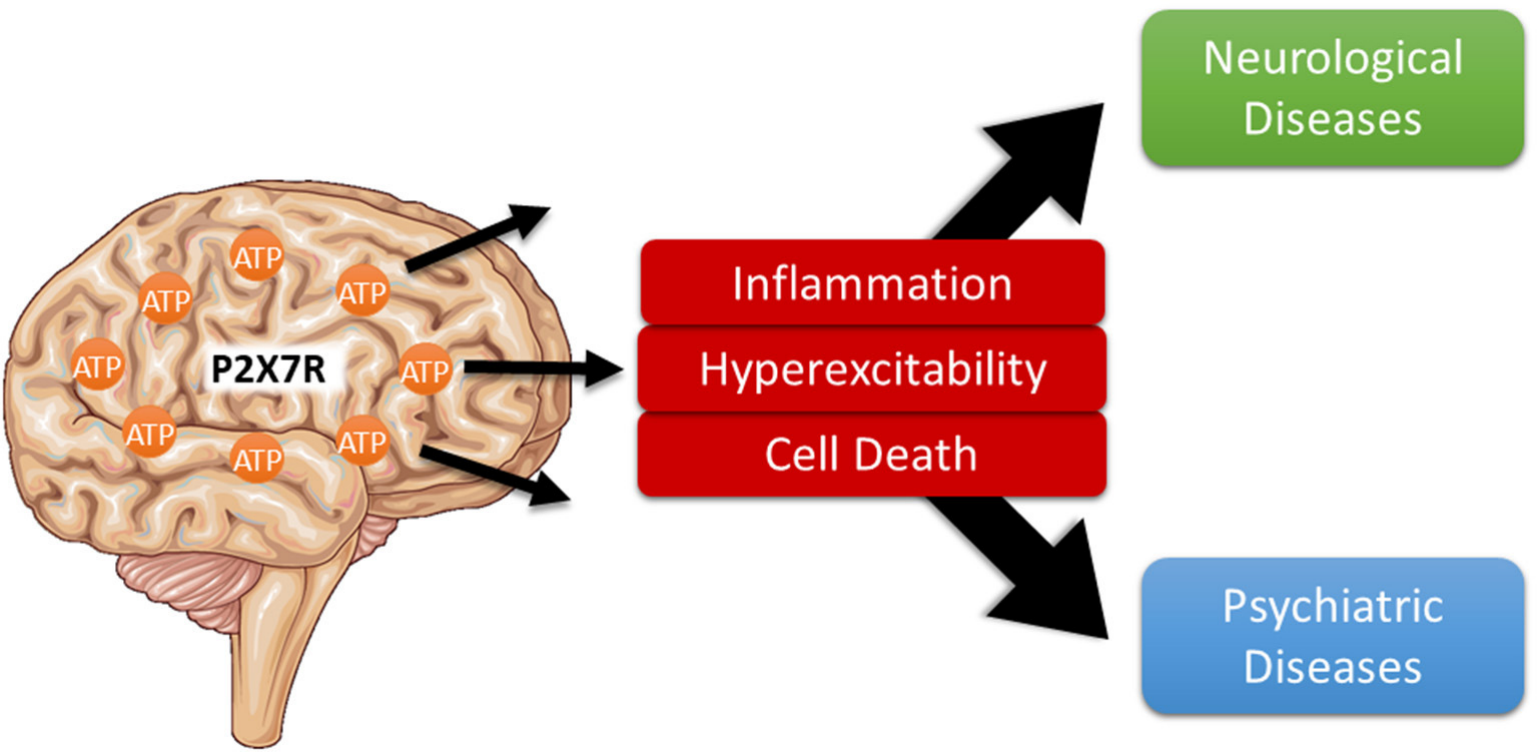
P2X7 receptor activation as shared pathological pathway in brain diseases. Usually found at low extracellular concentrations, ATP levels strongly increase during noxious conditions in the brain. Once released, ATP activates specific purinergic receptors including the ionotropic P2X7 receptor which, in turn, contribute to multiple pathological processes shared among several neurological and psychiatric brain diseases including neuroinflammation, increased hyperexcitability, and neurodegeneration thereby driving disease progression.

This Research Topic comprises 11 articles containing five reviews, three original research articles, two brief research reports and one hypothesis/theory article that summarize P2X7 receptor research in different brain diseases and provide up-to date and focused insights into the role of the P2X7 receptor and its potential as a drug target. In the review article written by Francistiová et al. novel findings of the role the P2X7 receptor in Alzheimer's disease are highlighted including data from animal models and humans. Ruiz-Ruiz et al. provided a review about the role of the P2X7 receptor in amyotrophic lateral sclerosis with a particular focus on how the mitigation of neuroinflammation *via* P2X7 receptor blockade may lead to a higher motoneuron survival in patients. Cisneros-Mejorado et al. reviewed the latest findings on the P2X7 receptor in cerebro-vascular disease and Illes et al. contributed an up-to date summary of the P2X7 receptor involvement in major depression and bipolar disorders focussing on possible contributions from both astrocytes and microglia. Finally, Andrejew et al. discusses the molecular mechanisms underlying P2X7 receptor-mediated signaling in neurodegenerative diseases, psychiatric disorders, and brain tumors and highlights the recent advances in the development of P2X7 receptor antagonists. The original research article by Ollà et al. shows for the first time increased P2X7 receptor protein levels in the brain of patients with Huntington's disease supporting the idea that the P2X7 receptor provides a possible therapeutic target in this devastating disease. Interestingly, the observed increases in P2X7 receptor expression in patients are accompanied by disease-specific alterations in the expression of different P2X7 receptor splice variants. Using a mouse model of intraperitoneal phencyclidine (PCP), Calovi et al. support the idea of the P2X7 receptor as a potential therapeutic target in schizophrenia. Using mouse models with either increased or decreased P2X7 receptor expression, the authors show that the P2X7 receptor drives PCP-mediated effects including changes in behavior, basal dopamine concentrations, layer-specific neuronal activation, intrinsic excitability of neurons and the interaction of microglia with hyperactive neurons. In a mouse model of status epilepticus, Conte et al. show for the first time how P2X7 receptor signaling impacts on the expression profile of microRNAs in the brain during normal physiology and following prolonged damaging seizures (i.e., status epilepticus) and suggest a novel pathway of how the P2X7 receptor might contribute to the gene expression landscape during both the maintenance of normal cellular homeostasis and pathological processes. In one of the brief research reports Bibič and Stokes tested the hypothesis of the P2X7 receptor being activated *via* amyloid β peptides. Performing different *in vitro* studies, the authors found, however, no evidence that amyloid β peptides act as agonists of the P2X7 receptor and conclude that amyloid β peptides simply mimic features of P2X7 receptor activation. In another brief research article, Rissiek et al. evaluated the susceptibility of astrocytes and microglia to cell death induced *via* P2X7 receptor activation through its ADP-ribosylation caused by NAD^+^. Their data show that treatment of microglia or astrocytes with NAD^+^ resulted neither in the activation of the P2X7 receptor nor induction of cell death and explain these results with the finding that astrocytes and microglia preferentially express the ADP-ribosylation-insensitive P2X7a splice variant. Finally, Sanz et al. found a correlation of certain P2X7 receptor single nucleotide polymorphisms (SNPs) with age and hypothesize that these SNPs may promote an anti-inflammatory phenotype, thereby extending life expectancy among the European and North-American Caucasian population.

In summary, this Research Topic provides a state of-the art description of P2X7 receptor research in the CNS, further supporting the concept of P2X7 activation being a shared pathological pathway among a broad spectrum of brain diseases.

## Author Contributions

All authors listed have made a substantial, direct and intellectual contribution to the work, and approved it for publication.

## Conflict of Interest

The authors declare that the research was conducted in the absence of any commercial or financial relationships that could be construed as a potential conflict of interest.

## References

[B1] Burnstock G. (2020). Introduction to purinergic signalling in the brain. Adv. Exp. Med. Biol. 1202, 1–12. 10.1007/978-3-030-30651-9_1 32034706

[B2] Cepeda-Prado E. Popp S. Khan U. Stefanov D. Rodríguez J. Menalled L. B.. (2012). R6/2 Huntington's disease mice develop early and progressive abnormal brain metabolism and seizures. J. Neurosci. 32, 6456–6467. 10.1523/JNEUROSCI.0388-12.2012 22573668 PMC3374973

[B3] Di Virgilio F. Dal Ben D. Sarti A. C. Giuliani A. L. Falzoni S. (2017). The P2X7 receptor in infection and inflammation. Immunity 47, 15–31. 10.1016/j.immuni.2017.06.020 28723547

[B4] Jimenez-Mateos E. M. Smith J. Nicke A. Engel T. (2019). Regulation of P2X7 receptor expression and function in the brain. Brain Res. Bull. 151, 153–163. 10.1016/j.brainresbull.2018.12.008 30593878

[B5] Kanner A. M. (2012). Can neurobiological pathogenic mechanisms of depression facilitate the development of seizure disorders? Lancet Neurol. 11, 1093–1102. 10.1016/S1474-4422(12)70201-6 23021976

[B6] Kopp R. Krautloher A. Ramírez-Fernández A. Nicke A. (2019). P2X7 interactions and signaling - making head or tail of it. Front. Mol. Neurosci. 12:183. 10.3389/fnmol.2019.00183 31440138 PMC6693442

[B7] Nakahara S. Adachi M. Ito H. Matsumoto M. Tajinda K. van Erp T. G. M. (2018). Hippocampal pathophysiology: commonality shared by temporal lobe epilepsy and psychiatric disorders. Neurosci. J. 2018:4852359. 10.1155/2018/4852359 29610762 PMC5828345

[B8] Palop J. J. Mucke L. (2010). Amyloid-beta-induced neuronal dysfunction in Alzheimer's disease: from synapses toward neural networks. Nat. Neurosci. 13, 812–818. 10.1038/nn.2583 20581818 PMC3072750

[B9] Robel S. Sontheimer H. (2016). Glia as drivers of abnormal neuronal activity. Nat. Neurosci. 19, 28–33. 10.1038/nn.4184 26713746 PMC4966160

[B10] Sperlagh B. Illes P. (2014). P2X7 receptor: an emerging target in central nervous system diseases. Trends Pharmacol. Sci. 35, 537–547. 10.1016/j.tips.2014.08.002 25223574

